# Combined transcriptome and metabolome reveal glutathione metabolism plays a critical role in resistance to salinity in rice landraces HD961

**DOI:** 10.3389/fpls.2022.952595

**Published:** 2022-09-07

**Authors:** Shan Yang, Mengshuang Liu, Na Chu, Guanxiu Chen, Panpan Wang, Junjie Mo, Haifeng Guo, Jianghuan Xu, Hongkai Zhou

**Affiliations:** ^1^College of Coastal Agricultural Sciences, South China Branch of National Saline-Alkali Tolerant Rice Technology Innovation Center, Guangdong Ocean University, Zhanjiang, China; ^2^National Engineering Research Center for Sugarcane, Fujian Agriculture and Forestry University, Fuzhou, China

**Keywords:** *Oryza sativa*, salt stress, glutathione, transcriptome, metabolome

## Abstract

Rice (*Oryza sativa*) is one of the most important food crops around the world, which is sensitive to salt stress, especially in the seedling and booting stage. HD961 is a salt-tolerant rice landrace that grows along coastal beaches and has disease and insect pest resistance, salt tolerance, and vigorous growth characteristics. We performed a combined transcriptome and metabolome analysis to clarify salinity resistance mechanisms in cultivar HD961, which has adapted to salinity soil at the early seedling stage. The results showed that the growth and antioxidant capacity of HD961 were stronger than 9311 under salt stress (SS). Transcriptomic analysis showed that a total of 6,145, 3,309, 1,819, and 1,296 differentially expressed genes (DEGs) were identified in the groups of TH60 (control group vs. 60 mM group of HD961 for transcriptome), TH120 (control group vs. 120 mM group of HD961 for transcriptome), T60 (control group vs. 60 mM group of 9311 for transcriptome), and T120 (control group vs. 120 mM group of 9311 for transcriptome), respectively. Starch and sucrose metabolism and phenylpropanoid biosynthesis were shared in the four treatment groups based on a KEGG enrichment analysis of DEGs. In addition, alpha-linolenic acid metabolism, plant hormone signal transduction, plant-pathogen interaction, and fatty acid elongation were specific and significantly different in HD961. A total of 92, 158, 151, and 179 significantly regulated metabolites (SRMs) responded to SS in MH60 (control group vs. 60 mM group of HD961 for metabolome), MH120 (control group vs. 120 mM group of HD961 for metabolome), M60 (control group vs. 60 mM group of 9311 for metabolome), and M120 (control group vs. 120 mM group of 9311 for metabolome), respectively. The KEGG analysis showed that eight common metabolic pathways were identified in the four treatment groups, of which biosynthesis of amino acids was the most significant. Three specific metabolic pathways were identified in the HD961, including glutathione metabolism, ascorbate and aldarate metabolism, and pantothenate and CoA biosynthesis. Integrative analysis between the transcriptome and metabolome showed that glutathione metabolism was specific and significantly affected under SS in HD961. A total of seven SRMs and 48 DEGs and four SRMs and 15 DEGs were identified in the glutathione metabolism pathway in HD961 and 9311, respectively. The Pearson correlation analysis showed a significant correlation between reduced glutathione and 16 genes (12 upregulated and four downregulated genes), suggesting these genes could be candidates as salt-tolerance regulation genes. Collectively, our data show that glutathione metabolism plays a critical role in response to SS in rice. Moreover, the stronger regulative ability of related common genes and metabolites might contribute to salt resistance in HD961.

## Introduction

As the human population continues to grow, the demand for cereals is increasing (Tilman et al., 2011). While breeders are trying to improve rice yield, the area of soil salinization is constantly expanding, seriously restricting the improvement in rice yield. Thus, collecting salt-tolerant rice germplasm and cultivating salt-tolerant rice varieties is particularly important. The HD961 variant, commonly known as “seawater rice,” is salt-tolerant landrace rice that grows along coastal beaches or low-lying saline fields often flooded with seawater in Zhanjiang, China. It has the characteristics of diseases and insect pest resistance, salt tolerance, and vigorous growth. Therefore, exploring the mechanisms of salt tolerance in HD961 will aid in developing new salt-tolerant varieties.

Water stress, ion toxicity, nutritional disorders, oxidative stress, changes in metabolic processes, and membrane disorganization are induced in plants under salt stress (SS) (Demidchik et al., 2010; Flowers and Colmer, 2015). These effects result in plant growth inhibition, developmental changes, metabolic adaptations, and death (Liang et al., 2018). Therefore, plants have evolved various mechanisms to develop salt tolerance, including osmotic adjustment, signal transduction, ion sequestration or exclusion, molecular regulation, and adaptive responses (van Zelm et al., 2020). Many osmoprotectants, such as proline, glycine, betaine, and soluble sugar, are induced by SS and consequently increase rapidly (Kishor et al., 1995; Garg et al., 2002; Taji et al., 2002). The rapid development of gene sequencing technologies in recent years has led to the identification of differentially expressed genes (DEGs) in response to SS based on transcriptome analysis (Fu et al., 2019; Jahan et al., 2021). Many genes, including transcription factors (TFs), osmoprotectant genes, and ion transporter/channel genes, have subsequently been cloned in rice for functional analysis, including *OsMYB2*, *OsbZIP71*, *OsCMO*, and *OsHKT1;5* (Luo et al., 2012; Yang et al., 2012; Liu et al., 2014; Kobayashi et al., 2017). The mechanisms of plant salt tolerance involve complex regulatory networks; therefore, it is necessary to understand the relationship between genes and downstream metabolites.

Metabolomics has been widely used to study complex metabolites under biotic and abiotic stress in plants, providing insight into extensive metabolic data and daedal metabolic pathways. Metabolomics can be divided into non-targeted and targeted metabolomics (Saito and Matsuda, 2010; Luo et al., 2018; Ribbenstedt et al., 2018). Widely targeted metabolomics represents the next generation of metabolome technology, which combines the broad spectrum of non-targeted metabolome detection with the accuracy of targeted metabolome detection. It mainly collects data through multiple reaction monitoring technologies and establishes a metabolite database to achieve an accurate qualitative and quantitative analysis of metabolites (Luo et al., 2018). Li et al. (2021) found that flavonoid biosynthesis may represent the main resistance mechanism for *Zanthoxylum bungeanum* against stem canker based on combined transcriptome and metabolome analyses. Moreover, the metabolites of *Jerusalem artichoke* involved in glycolysis, phenolic metabolism, tricarboxylic cycle, glutamate-mediated proline biosynthesis, urea cycle, amino acid metabolism, unsaturated fatty acid biosynthesis, and the met salvage pathway respond to drought stress (Zhao et al., 2021). Nine salt-tolerance biomarkers were identified by comparing metabolome data of two cultivars of hulless barley. The ABA signaling pathway was shown to be essential during the SS response (Wang et al., 2019b). A comparison of different metabolites in cultivar FL478 (salt-tolerance) and cultivar IR64 (salt-sensitive) at the seedling stage under SS found that sugars and amino acids were more obviously increased in FL478 than IR64, but organic acids of FL478 were clearly decreased compared to IR64 (Zhao et al., 2014). The metabolism of amino acids, sugars, linoleic acid, and wax biosynthesis was highly correlated with melatonin-mediated SS responses in rice (Xie et al., 2020). Therefore, widely targeted metabolomics is a mature detection technology that can be applied in this study.

In this study, RNA sequencing and widely targeted metabolomics were performed for rice cultivars HD961 (salt-tolerant) and 9311 (salt-sensitive) at the three-leaf stage with different concentrations of NaCl stress treatment (0, 60, and 120 mM) for 0 and 7 days, respectively. We compared and analyzed the transcriptome and metabolome between HD961 and 9311 in response to SS at the seedling stage. Here, we explored gene-gene, metabolome-metabolome, and gene-metabolome regulatory network relationships in the SS response in rice. Based on these data, the molecular regulation mechanism of salt tolerance was elucidated in the new salt-tolerant rice cultivar HD961. Related regulatory genes and metabolites in the metabolic pathway were found to improve the salt tolerance of rice. Our findings enrich the molecular regulation theory of salt tolerance in rice and provide a theoretical basis for salt tolerance rice breeding.

## Materials and methods

### Plant materials and cultivation

The Indica rice cultivars HD961 (salt-tolerant, red rice) and 9311 (salt-sensitive and white rice) were used in this study. Plump seeds were picked from the seeds of HD961 and 9311, which were surface-sterilized with 30% (v/v) H_2_O_2_ for 30 min, rinsed thoroughly with ddH_2_O and sown in a plastic tray (41.5 cm × 30.5 cm × 2 cm) covered with a layer of absorbent filter paper. The trays were then placed in the dark incubator at 28°C for 5 days, and the filter paper was kept moist with ddH_2_O. Seedlings with consistent growth vigor were transferred to a 96-well black hydroponic box filled with Yoshida nutrient solution of rice (Coolaber, Beijing, China) and cultivated in an artificial climate chamber with 65% relative humidity under an 18 h/6 h light/dark (light intensity ∼ 16,000 LUX) cycle at 26°C. The nutrient solution was renewed every 3 days.

### Salt stress treatment

The SS experiment used three NaCl concentrations: 0 mM (control group), 60 mM, and 120 mM, respectively. When the rice seedlings were at the stage of three leaves, plants with the same growth vigor were selected as experimental materials for a 7 days SS treatment. After the SS treatment, a phenotypic measurement was performed. The aerial part of the rice seedlings (a mix of stems and leaves) was collected for relevant omics and physiological analyses. All samples (18 samples) were frozen in liquid nitrogen and transferred to −80°C for storage. Three biological replicates were performed for each sample in this study.

### Measurement of phenotypic and physiologically related parameters

The phenotypic measurements included plant height, total root length, dry weight of the aerial part for each plant, and dry weight of the root system for each plant. Plant height was measured for 20 plants of each cultivar using a ruler. The 20 plants were cut into aerial and root sections. The root systems of the 20 plants were scanned using a root scanner (Microtek Scanmaker i800 Plus, China), and the root length was analyzed using the bundled software from the root scanner. After root system scanning, the aerial and root sections of 20 plants were put into two envelopes and incubated at 105°C for 30 min, followed by 85°C until they achieved a stable weight. Free proline and soluble sugar concentrations were determined using the acid ninhydrin reagent and anthrone methods, respectively (Moustakas et al., 2011). The H_2_O_2_ content and superoxide dismutase (SOD) activity were determined using Dongsansuk’s method (Dongsansuk et al., 2021).

### RNA sequencing and transcriptomics analysis

The total RNA from 18 samples was extracted using TRIzol reagent (Invitrogen, Carlsbad, CA, United States) according to the standard protocol. The cDNA library was then constructed and sequenced on an Illumina HiSeq X-ten platform at Wuhan Metware Biotechnology Co., Ltd. (Wuhan, China), and the length of the generated reads was 150 bp paired-end (PE 150). To obtain clear reads, the low-quality reads, adapter sequences, and sequences with more than 10% poly-N in the raw reads were filtered using fastp 0.21.0 (Chen et al., 2018). The Q20, Q30, and GC content of each sample were calculated. Clear reads from each sample were mapped to the Nipponbare (*Oryza sativa* ssp. indica, ASM465v1^1^) reference genome using HISAT2 (Kim et al., 2015). Raw counts of genes were performed using feature counts (Liao et al., 2014). The differentially expressed genes (DEGs) between two samples were identified using DESeq2 with the parameters of | log_2_fold change| ≥ 1 and false discovery rate (FDR) < 0.05 (Love et al., 2014). The functions of DEGs were annotated using the KEGG and GO databases, whereby the KEGG pathway analysis of DEGs was performed using BLAST software (Camacho et al., 2009). KEGG enrichment was analyzed using KOBAS 2.0 software with a *p*-value < 0.05 (Mao et al., 2005; Xie et al., 2011), and the GO analysis of DEGs was carried out using the R package cluster Profiler (Yu et al., 2012). The transcription factors (TFs) were predicted using iTAK software (Zheng et al., 2016) with the PlnTFDB (Pérez-Rodríguez et al., 2010) and PlantTFDB (Jin et al., 2014) databases among the DEGs. Moreover, the samples were named as TR_HD961_0 (control group of HD961 for transcriptome), TR_HD961_60 (60 mM group of HD961 for transcriptome), TR_HD961_120 (120 mM group of HD961 for transcriptome), TR_9311_0 (control group of 9311 for transcriptome), TR_9311_60 (60 mM group of 9311 for transcriptome), and TR_9311_120 (120 mM group of 9311 for transcriptome). TH60, TH120, T60, and T120 were denoted as groups TR_HD961_0 vs. TR_HD961_60, TR_HD961_0 vs. TR_HD961_120, TR_9311_0 vs. TR_9311_60, and TR_9311_0 vs. TR_9311_120, respectively.

### Real-time quantitative PCR

To confirm the accuracy of RNA-Seq, 12 candidate DEGs were verified by RT-qPCR. Based on the coding gene sequences, the primers of RT-qPCR were designed using primer premier 6.0 software, and β-actin was selected as the internal control gene (Kim et al., 2003) (Supplementary Table 1). The SYBR^®^ Green Premix Pro Taq HS qPCR Kit (Accurate Biological Engineering LTD, Hunan, China) was used for the RT-qPCR assay. A 20 μl of reaction solution contained 10 μl 2 × SYBR Green Pro Taq HS Premix, 0.5 μl of primer F (10 μM), 0.5 μl of primer R (10 μM), 1 μl of cDNA, and 8 μl of nuclease-free water. The qPCR reactions involved denaturation at 95°C for 30 s, followed by 40 cycles of 5 s at 95°C and 30 s at 60°C. The RT-qPCR assays were carried out using the Bio-Rad CFX Connect Real-time System. The qPCR data were analyzed using the 2^–ΔΔCt^ quantitative method to determine differences in gene expression (Livak and Schmittgen, 2001). This study used three independent biological replicates and three technological replicates for each sample.

### Sample preparation and extraction for widely targeted metabolomics

The sample preparation, extraction, identification, and quantification of the widely targeted metabolome was performed by Wuhan Metware Biotechnology Co., Ltd.^2^. Three biological replicates were performed in this study. The samples were named as follows: ME_HD961_0 (control group of HD961 for metabolome), ME_HD961_60 (60 mM group of HD961 for metabolome), ME_HD961_120 (120 mM group of HD961 for metabolome), ME_9311_0 (control group of 9311 for metabolome), ME_9311_60 (60 mM group of 9311 for metabolome), and ME_9311_120 (120 mM group of 9311 for metabolome). MH60, MH120, M60, and M120 were denoted as groups ME_HD961_0 vs. ME_HD961_60, ME_HD961_0 vs. ME_HD961_120, ME_9311_0 vs. ME_9311_60, and ME_9311_0 vs. ME_9311_120, respectively.

Biological samples were freeze-dried using a vacuum freeze dryer (Scientz-100F). The freeze-dried samples were crushed using a mixer mill (MM 400, Retsch) with a zirconia bead for 1.5 min at 30 Hz. Next, 100 mg of lyophilized powder was dissolved with 1.2 ml 70% methanol solution, vortexed for 30 s every 30 min for a total of six times, and then placed at 4°C overnight. Following centrifugation at 12,000 rpm for 10 min, the extracts were filtered (SCAA-104, 0.22 μm pore size; ANPEL, Shanghai, China) before UPLC-MS/MS analysis.

### UPLC conditions

The sample extracts were analyzed using a UPLC-ESI-MS/MS system (UPLC, SHIMADZU Nexera X2; MS, Applied Biosystems 4500 Q TRAP). The analytical conditions were as follows: UPLC: column, Agilent SB-C18 (1.8 μm, 2.1 mm × 100 mm); the mobile phase consisted of solvent A, pure water with 0.1% formic acid, and solvent B, acetonitrile with 0.1% formic acid. Sample measurements were performed with a gradient program that employed starting conditions of 95% A and 5% B. Within 9 min, a linear gradient was programmed to 5% A and 95% B, and a composition of 5% A and 95% B was maintained for 1 min. Subsequently, a composition of 95% A and 5.0% B was adjusted within 1.10 min and maintained for 2.9 min. The flow velocity was set at 0.35 ml per minute, the column oven was set to 40°C, and the injection volume was set to 4 μl. The effluent was alternatively connected to an ESI-triple quadrupole-linear ion trap (QTRAP)-MS.

### ESI-Q TRAP-MS/MS

The LIT and triple quadrupole (QQQ) scans were acquired on a triple quadrupole-linear ion trap mass spectrometer (Q TRAP), the AB4500 Q TRAP UPLC/MS/MS System, equipped with an ESI Turbo Ion-Spray interface that was operated in positive and negative ion mode and controlled by Analyst 1.6.3 software (AB Sciex). The ESI source operation parameters were as follows: an ion source, turbo spray; source temperature, 550°C; ion spray voltage (IS), 5,500 V (positive ion mode)/−4,500 V (negative ion mode); ion source gas I (GSI), gas II (GSII), and curtain gas (CUR) were set at 50, 60, and 25 psi, respectively; and the collision-activated dissociation (CAD) was set at high. Instrument tuning and mass calibration were performed with 10 and 100 μM polypropylene glycol solutions in QQQ and LIT modes, respectively. QQQ scans were acquired as MRM experiments with collision gas (nitrogen) set to medium. DP and CE for individual MRM transitions were conducted with further DP and CE optimization. A specific set of MRM transitions were monitored for each period according to the metabolites eluted within this period.

### Data analysis of metabolomics

The hierarchical cluster analysis (HCA) results of samples and metabolites were presented as heatmaps with dendrograms, while Pearson correlation coefficients (PCC) between samples were calculated by the cor function in R and presented as only heatmaps. Both HCA and PCC were carried out by the R package pheatmap. For HCA, normalized signal intensities of metabolites (unit variance scaling) were visualized as color spectra. Principal component analysis (PCA) was performed using the statistics function prcomp within R^3^. The data were unit variance scaled before PCA.

Significantly regulated metabolites (SRMs) between groups were determined by VIP ≥ 1 and absolute Log_2_FC (fold change) ≥ 1. VIP values were extracted from the OPLS-DA results, which also contained score plots and permutation plots, and were generated using the R package MetaboAnalystR. The data were log-transformed (log_2_) and mean centered before OPLS-DA. To avoid overfitting, a permutation test (200 permutations) was performed.

Identified metabolites were annotated using the KEGG compound database^4^, and annotated metabolites were then mapped to the KEGG pathway database^5^. The pathways with SRMs mapped were then fed into the metabolite set enrichment analysis (MSEA), and their significance was determined using the hypergeometric test’s *p*-values.

### Statistical analysis

Duncan’s multiple comparison method was conducted using Statistical Product and Service Solutions software (IBM SPSS 19.0) to assess the differences in phenotypic and physiological indices at three different levels of salinity. Statistical significance was set at *p* < 0.05. The bar diagram was drawn using Microsoft Office Excel 2016. The Pearson correlation analysis and gene-metabolite correlation network diagram between DEGs and SRMs in the glutathione pathway of HD961 were conducted using a tool of advanced correlation network graph in the Metware cloud platform (the threshold for association analysis > 0.8, *p* < 0.05^6^).

## Results

### Analysis of morphological response to salt stress

The HD961 cultivar showed better plant height, total root length, dry weight of the aerial part, and dry weight of roots than the cultivar 9311 after 7 days of SS treatment (Supplementary Figure 1). The plant height and root length of HD961 and 9311 decreased as the NaCl concentration increased (Supplementary Figures 1A,B). Compared with 0 mM NaCl, the dry weight of the aerial part and roots were slightly increased at 60 mM and 120 mM in HD961, but decreased for 9311 (Supplementary Figures 1C,D). In addition, the biomass of HD961 was significantly greater than 9311 after 7 days of SS treatment (Supplementary Figure 2). Therefore, the performance of HD961 was better than 9311 under NaCl stress.

### Analysis of the physiological response to salt stress

**FIGURE 1:**
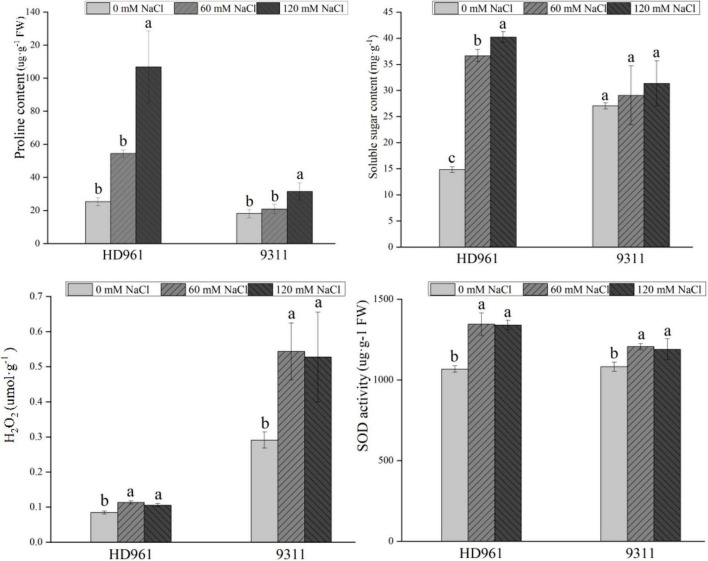
Physiologic parameters of HD961 and 9311 after 7-day salt stress treatment. The values of the error bars are plus and minus standard deviation values. The different letters denote that it is at the significance level among different concentrations of the same cultivar, *p* < 0.05.

The content of H_2_O_2_, proline, soluble sugar, and SOD activity were significantly increased in HD961 and 9311 cultivars under NaCl stress, except for the soluble sugar content of 9311 (Figure 1). The H_2_O_2_ content of HD961 was less than 9311, but the proline content, soluble sugar content, and SOD activity of HD961 were greater than those of 9311 under SS (Figure 1). Therefore, HD961 has a stronger osmotic adjustment ability and antioxidant capability than 9311 when the plants are under SS.

### Transcriptome analysis

To investigate the transcriptome response to SS in rice, 18 cDNA libraries were sequenced using Illumina platforms. After quality control, there were 44,784,392 to 60,082,654 clean reads in HD961 and 44,686,470 to 56,907,604 clean reads in 9311 (Supplementary Table 2). The Q20 and Q30 of all libraries were more than 97.77 and 94.00%, respectively (Supplementary Table 2). Twelve genes were chosen for qRT-PCR to validate the RNA-seq data, including *LOC_Os01g19330*, *LOC_Os01g46760*, *LOC_Os01g51690*, *LOC_Os01g68460*, *LOC_Os03g44270*, *LOC_Os04g33920*, *LOC_Os05g04870*, *LOC_Os06g02040*, *LOC_Os06g09688*, *LOC_Os07g19320*, *LOC_Os08g36310*, and *LOC_Os10g08474*. The results showed that the expression profiles of these genes were consistent with those results of RNA-seq, suggesting that the RNA-seq data were accurate and reliable (Supplementary Figure 3).

**FIGURE 2:**
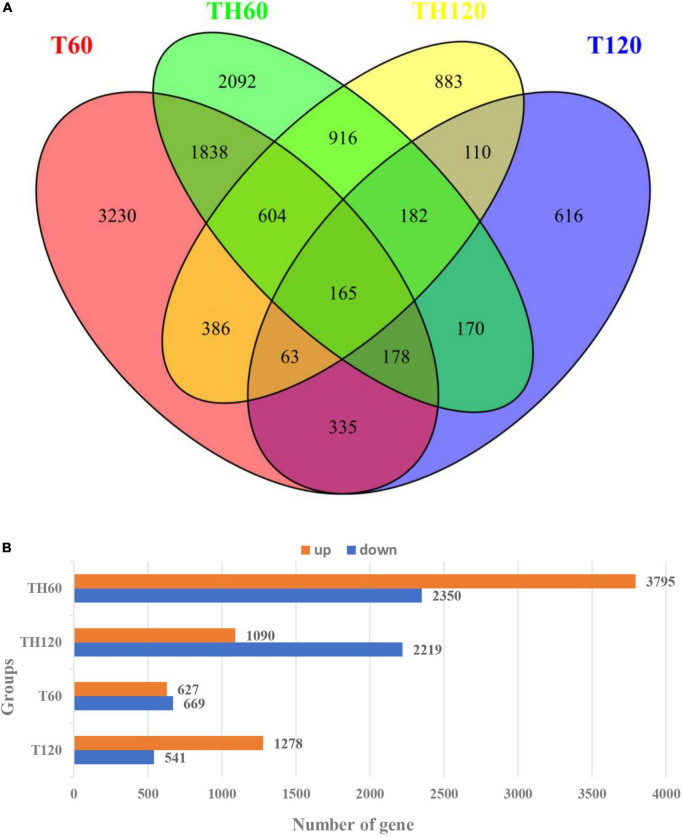
All differentially expressed genes (DEGs) at 60 mM and 120 mM NaCl treatment. **(A)** The DEGs Venn diagram of HD961and 9311. **(B)** The up and downregulated DEGs of HD961 and 9311.

A total of 6,145, 3,309, 1,819, and 1,296 DEGs were identified in the TH60, TH120, T60, and T120 groups, respectively, of which 1,867 common DEGs were expressed in both TH60 and TH120, and 490 common DEGs were expressed in T60 and T120 (Figure 2A). In addition, 2,784 and 520 common DEGs were expressed in two cultivars at 60 and 120 mM, respectively (Figure 2A). The 3,231, 2,093, 883, and 616 DEGs were expressed exclusively in groups T60, TH60, TH120, and T120 (Figure 2A). These results showed that the number of DEGs in HD961 was greater than in 9311. Compared with the 120 mM treatment group, the DEGs were more responsive at 60 mM in both cultivars. There were 3,795 up-DEGs and 2,350 down-DEGs, as well as 1,090 up-DEGs and 2,219 down-DEGs in TH60 and TH120, respectively. In addition, there were 627 up-DEGs and 669 down-DEGs, as well as 1,278 up-DEGs and 541 down-DEGs in T60 and T120, respectively (Figure 2B). A heatmap for all DEGs under different concentrations of SS was generated after RNA-seq analysis. It showed that the gene expression patterns of the two cultivars were clearly different under SS (Supplementary Figure 4). The DEGs of HD961 exhibited clearer differences under the tested NaCl concentrations than 9311.

**FIGURE 3:**
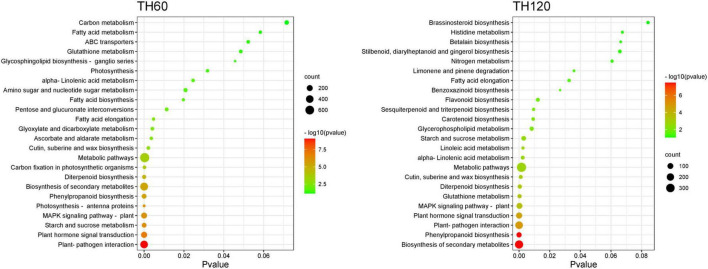
Top 24 pathways of KEGG enrichment of DEGs in HD961.

**FIGURE 4:**
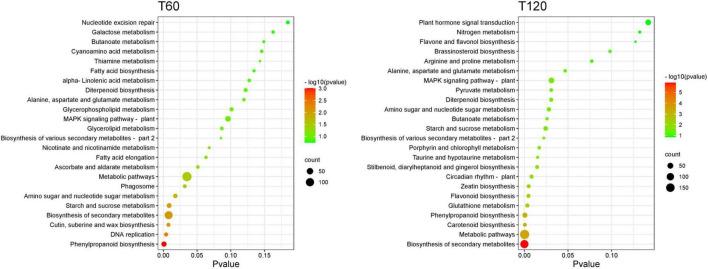
Top 24 pathways of KEGG enrichment of DEGs in 9311.

The KEGG pathway enrichment analysis of DEGs showed that 134, 121, 100, and 108 KEGG pathways were enriched in the TH60, TH120, T60, and T120 groups, respectively (Supplementary Tables 3–6). Moreover, 21, 19, 8, and 19 KEGG pathways were significantly enriched in the TH60, TH120, T60, and T120 groups, respectively (Figure 3). The Top 5 KEGG pathways of TH60 were increased considerably by 404, 211, 89, 187, and 12 DEGs. They included plant-pathogen interaction, plant hormone signal transduction, starch and sucrose metabolism, MAPK signaling pathway-plant, and photosynthesis-antenna proteins, respectively (Figure 3 and Supplementary Table 3). In addition, 66, 216, 117, 101, and 27 DEGs were significantly enriched for the top 5 KEGG pathways in TH120, namely phenylpropanoid biosynthesis, plant–pathogen interaction, plant hormone signal transduction, MAPK signaling pathway-plant, and glutathione metabolism, respectively (Figure 3 and Supplementary Table 4). For the T60 group, 24, 10, 9, 19, and 14 DEGs were significantly enriched for the top 5 KEGG pathways: phenylpropanoid biosynthesis, DNA replication, cutin, suberine, and wax biosynthesis, starch, and sucrose metabolism, and amino sugar and nucleotide sugar metabolism, respectively (Figure 4 and Supplementary Table 5). 10, 29, 14, 13, and 11 DEGs were significantly enriched to carotenoid biosynthesis, phenylpropanoid biosynthesis, glutathione metabolism, flavonoid biosynthesis, and zeatin biosynthesis in the T120, respectively (Figure 4 and Supplementary Table 6). Moreover, two KEGG pathways were shared and significantly different in four groups (TH60, TH120, T60, and T120; *p* < 0.05), including starch and sucrose metabolism (89, 44, 19, and 21 DEGs) and phenylpropanoid biosynthesis (96, 66, 24, and 29 DEGs), respectively (Figures 3, 4 and Supplementary Figure 5). Alpha-linolenic acid metabolism (51 and 19 DEGs), plant hormone signal transduction (211 and 117 DEGs), plant-pathogen interaction (404 and 216 DEGs), and fatty acid elongation (20 and 11 DEGs) were specific KEGG pathways in both TH60 and TH120 (Figure 3 and Supplementary Figure 5). In the MAPK signaling pathway–plant (187, 101, and 44 DEGs), glutathione metabolism (33, 27, and 14 DEGs) and diterpenoid biosynthesis (52, 30, and 13 DEGs) were shared between the TH60, TH120, and T120 groups (Figures 3, 4 and Supplementary Figure 5).

### Metabolome analysis

**FIGURE 5:**
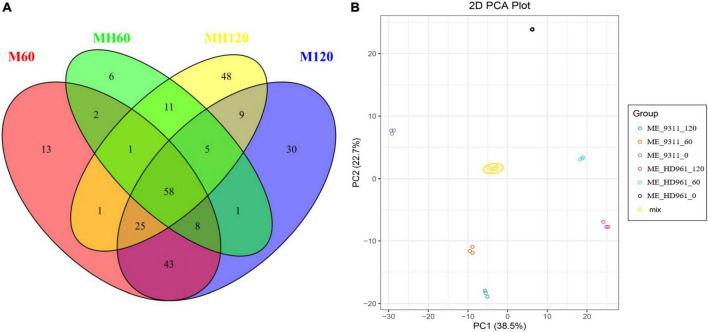
All SRMs at 60 mM and 120 mM NaCl treatment. **(A)** The SRMs Venn diagram of HD961and 9311. **(B)** PCA of metabolic data from different treatments in HD961 and 9311.

**FIGURE 6:**
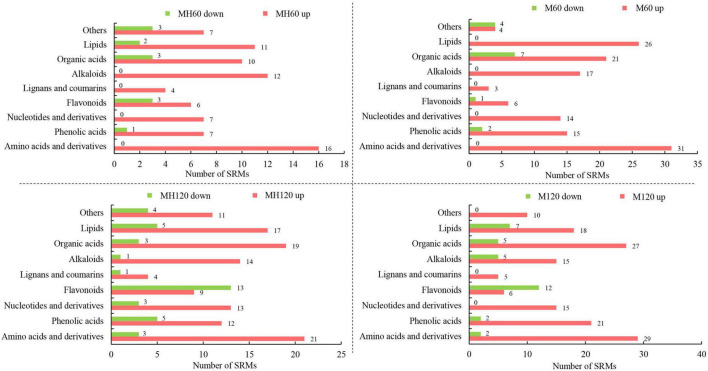
Significantly regulated metabolite (SRM) classification of HD961 and 9311.

The metabolites of HD961 and 9311 were analyzed to investigate the variation of the metabolomic profiles in response to SS using LC-MS/MS. A total of 92 (80 upregulated and 12 downregulated), 158 (120 upregulated and 38 downregulated), 151 (137 upregulated and 14 downregulated), and 179 (146 upregulated and 33 downregulated) SRMs accumulated in the MH60, MH120, M60, and M120 groups, respectively (Supplementary Figure 6). The 75 common SRMs accumulated in both MH60 and MH120, and the 134 common SRMs accumulated in both M60 and M120 (Figure 5A). The 69 and 97 common SRMs accumulated in two cultivars at 60 mM and 120 mM, respectively (Figure 5A). In addition, 13, 6, 48, and 30 SRMs were exclusively accumulated in the M60, MH60, MH120, and M120 groups (Figure 5A). These results show that as NaCl concentration increased, the number of SRMs also increased. Most of the SRMs were upregulated when the plants were subjected to SS. The PCA based on three QC samples (mix) and 18 test samples showed that 18 samples could separate clearly in the first two principal components, accounting for 61.2% of the total variability (Figure 5B). PCA1 and PCA2 accounted for 38.5% and 22.7% of the variability, respectively (Figure 5B). A heatmap of all metabolites showed significant differences in metabolites in HD961 and 9311 cultivars under NaCl stress (Supplementary Figure 7). These SRMs were classified into nine groups, of which the amino acids and derivatives (20%), organic acids (15.5%), lipids (14.8%), and alkaloids (12.3%) contained the majority of the upregulated SRMs. The flavonoids (25.7%), organic acids (24.5%), and lipids (12.8%) accounted for the largest proportion of downregulated SRMs (Figure 6 and Supplementary Figure 7). Therefore, amino acids and derivatives were the predominant upregulated SRMs, and the flavonoids and organic acids were the main downregulated SRMs in rice under NaCl stress.

**FIGURE 7:**
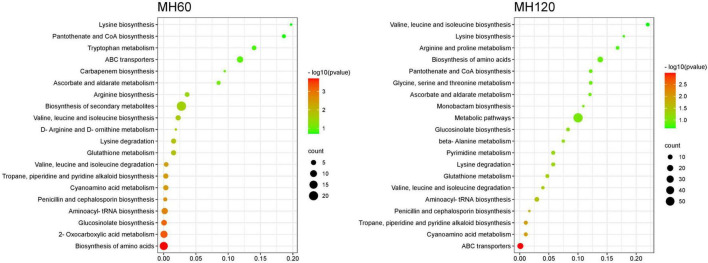
Top 20 pathways of KEGG enrichment of SRMs in HD961.

**FIGURE 8:**
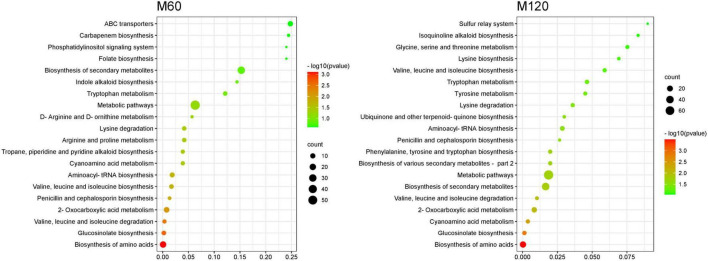
Top 20 pathways of KEGG enrichment of SRMs in 9311.

KEGG pathway enrichment analysis of SRMs showed that the top 20 KEGG pathways were similar in both HD961 and 9311 cultivars (Figures 7, 8). The most significant enrichments in MH60, MH120, M60, and M120 groups were biosynthesis of amino acids, ABC transporters, biosynthesis of amino acids, and biosynthesis of amino acids, respectively (Figures 7, 8 and Supplementary Tables 9–12). Therefore, the biosynthesis of amino acids was the most important metabolic pathway under NaCl stress in both cultivars. For example, L-cysteine and L-lysine-butanoic acid were the top two upregulated SRMs in the biosynthesis of amino acids (Supplementary Tables 8, 9). L-isoleucine, L-ornithine, L-valine, and L-proline were also upregulated SRMs. To visually describe the relationship among the top 20 KEGG enrichment pathways of the four groups, we created a Venn diagram (Supplementary Figure 8). The results showed that eight common metabolic pathways were identified in the four groups: biosynthesis of amino acids, glucosinolate biosynthesis, aminoacyl-tRNA biosynthesis, penicillin, and cephalosporin biosynthesis, cyanoamino acid metabolism, valine, leucine and isoleucine degradation, lysine degradation, and valine, leucine, and isoleucine biosynthesis (Supplementary Figure 8 and Supplementary Table 13). Of these common metabolic pathways, the cyanoamino acid metabolism, penicillin, and cephalosporin biosynthesis, aminoacyl-tRNA biosynthesis, and valine, leucine, and isoleucine degradation reached a statistically significant enrichment level in the four groups (*p* < 0.05) (Figures 7, 8). There were three specific metabolic pathways in the HD961 cultivar (glutathione metabolism, ascorbate and aldarate metabolism, and pantothenate and CoA biosynthesis) (Supplementary Figure 8 and Supplementary Table 13), of which glutathione metabolism was significantly enriched (*p* < 0.05). Therefore, glutathione metabolism should be explored in rice grown under SS.

### Integrative analysis between the transcriptome and metabolome

**FIGURE 9:**
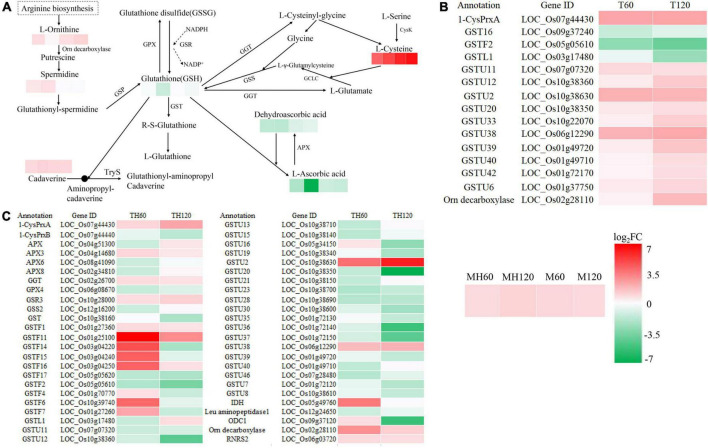
Analysis of DEGs and SRMs in the glutathione metabolic pathway. **(A)** The enriched SRMs of MH60, MH120, M60, and M120 in the glutathione metabolic pathway. Spermidine, GSH, and dehydroascorbic acid were not SRMs in the M60 and M120. **(B)** The enriched DEGs of T60 and T120 in the glutathione metabolic pathway. **(C)** The enriched DEGs of TH60 and TH120 in the glutathione metabolic pathway. |log_2_FC| denotes | log_2_fold change|.

**FIGURE 10:**
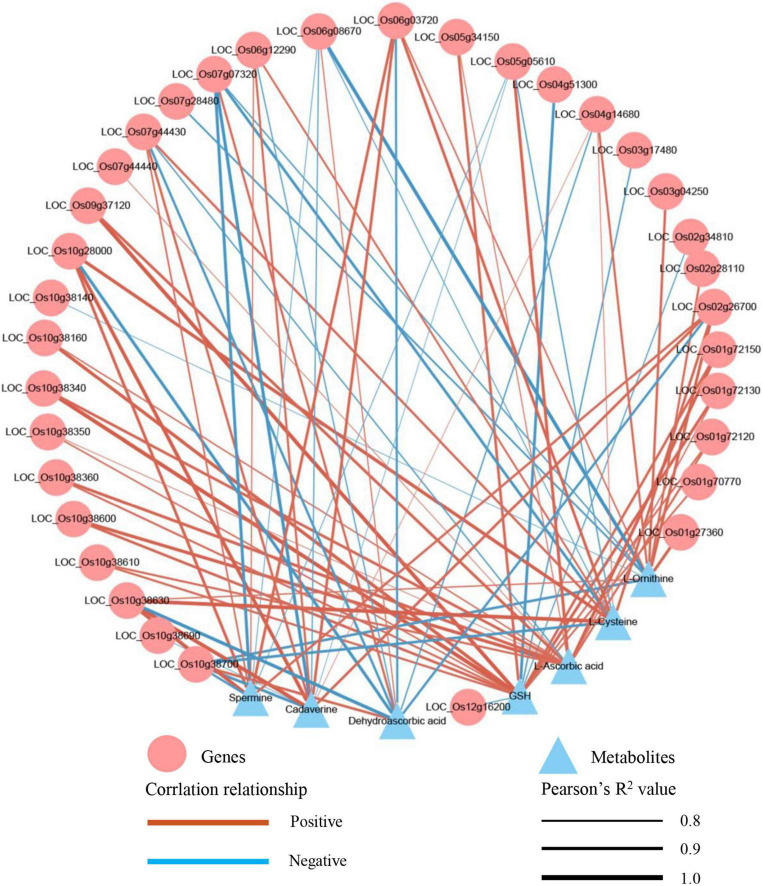
Correlation network diagram of DEGs and SRMs in the HD961.

To understand the relationship between DEGs and SRMs, we conducted an integrative analysis between the transcriptome and metabolome. Glutathione metabolism was the only shared and significantly enriched pathway in the TH60, MH60, TH120, and MH120 groups (Supplementary Tables 14, 15). However, no shared and significantly enriched pathways were observed in both T60, M60, T120, and M120 groups (Supplementary Tables 16, 17). Therefore, the specific glutathione metabolism represented a significantly affected metabolic pathway in the cultivar HD961 undergoing SS. In this pathway, seven SRMs and 48 DEGs were identified in the HD961, and four SRMs and 15 DEGs were identified in the 9311 undergoing SS (Figure 9). Compared with 9311, the HD961 cultivar had a stronger response to SS, and its regulation was more complex. Reduced glutathione (GSH), spermidine, and dehydroascorbic acid were specific SRMs in HD961 (Figure 9A). Moreover, L-ornithine (upregulated), cadaverine (downregulated), L-ascorbic acid (downregulated), and L-cysteine (upregulated) were the same SRMs in both cultivars, of which L-cysteine was the most active substance in response to SS (Figure 9A). GSH and dehydroascorbic acid decreased, while spermidine increased as NaCl concentration increased (Figure 9A). With regard to DEGs, most of the DEGs were glutathione *S*-transferase (GST), of which the ‘tau’ class GST (U represents tau) was the most active, followed by the ‘phi’ class GST (F represents phi) (Figures 9B,C). Therefore, the *GSTs* were clearly induced by NaCl stress. In HD961, the expression of ascorbate peroxidase 3 (*APX3*, *LOC_Os04g14680*), gamma-glutamyl transpeptidase (*GGT*, *LOC_Os02g26700*), and glutathione reductase (*GSR*, *LOC_Os10g28000*) were increased under SS. The expression of *APX6* (*LOC_Os08g41090*), *APX8* (*LOC_Os02g34810*), glutathione peroxidase (*GPX*, *LOC_Os06g08670*), and glutathione synthase (*GSS*, *LOC_Os12g16200*) first increased and then decreased as the concentration of NaCl increased. To explore the relationship between these SRMs and DEGs in glutathione metabolism, we conducted the Pearson correlation analysis. The results showed that 34 DEGs strongly correlated with 7 SRMs (*R*^2^ > 0.8 and *P* < 0.05) under NaCl stress (Figure 10). There was a significant correlation between the expression of genes (*LOC_Os10g38630*, *GSTU2*) and metabolite abundance (MA10039492, dehydroascorbic acid; pme0195, L-cysteine; mws0018, spermine; pme2527, L-ornithine; pme1841, cadaverine). Moreover, there were significant correlations between GSH and 16 genes (12 upregulated genes and four downregulated genes). For example, the correlation between the genes of *LOC_Os10g38340*, *LOC_Os09g37120*, *LOC_Os10g38160*, and *LOC_Os01g72130.* Further, the metabolite of GSH was extremely significant, with correlation coefficients of 0.993, 0.992, 0.963, and 0.939, respectively (Figure 10). Nevertheless, the correlation coefficient between GSH and the genes of *LOC_Os04g51300*, *LOC_Os03g17480*, *LOC_Os02g34810*, and *LOC_Os12g16200* were −0.934, −0.855, −0.837, and −0.836, respectively (Figure 10). Taken together, the correlation analysis revealed that specific genes were highly correlated with glutathione metabolites, indicating that these synthesis genes play a vital role in the synthesis of GSH under SS.

## Discussion

Soil salinity is a global problem that affects seed germination, crop growth, and production (Yang and Guo, 2018). It is becoming a serious problem worldwide due to excessive use of fertilizers, inappropriate crop irrigation, excessive plowing, and seawater intrusion in the coastal area (Kamran et al., 2020). Rice is an important cereal crop that feeds more than 50% of the world’s population but is sensitive to SS (Hussain et al., 2018). Fertile land is shrinking, the population is growing, and food shortages are becoming more prevalent. Therefore, there is a growing demand for salt-tolerant rice varieties. Understanding the growth, physiology, gene expression, and metabolic accumulation under SS is essential for developing salt-tolerant rice varieties.

The growth and development of plants are negatively affected by SS, resulting in several specific responses, including leaf rolling, reduced biomass, shorter plant height, and lower yields (Machado and Serralheiro, 2017; Razzaq et al., 2020). In this study, the plant height and root length of HD961 and 9311 cultivars were both decreased with an increasing concentration of NaCl. Therefore, the growth of HD961 and 9311 was restrained under SS. It is worth noting that the salt-tolerant cultivar HD961 exhibited greater biomass than the weak salt-tolerant cultivar 9311. With regard to physiology, several adaptive responses could occur in response to SS, including osmotic adjustment, regulation of ionic balance, nutritional balance, and reactive oxygen species (ROS) scavenging (Liu et al., 2022). In this study, the proline content, soluble sugar content, CAT activity, and SOD activity of HD961 were greater than 9311 under SS, and the O^2–^ and H_2_O_2_ concentrations of HD961 were less than 9311 under SS. Similar results have been shown in previous studies, including the upregulation of proline content, soluble sugar content, CAT activity, and SOD activity under SS (Liang et al., 2018; Nounjan and Theerakulpisut, 2021). Lee et al. (2013) found that the tolerance to SS in Pokkali is derived largely from constitutively maintained antioxidant enzymatic activities and induction of the antioxidant enzyme system. As a result, HD961 has a stronger ROS scavenging system than 9311, resulting in stronger salt resistance.

The gene expression of rice can be inducted by a diverse range of abiotic stresses, including SS (van Zelm et al., 2020; Liu et al., 2022). Our study performed an RNA-seq-based comparative transcriptome analysis to assess gene expression changes in rice in response to SS. A total of 6,145, 3,309, 1,819, and 1,296 DEGs were identified in the TH60, TH120, T60, and T120 groups, respectively, suggesting that more defense-responsive genes are activated in the salt-tolerant cultivar HD961 relative to the cultivar 9311. KEGG analysis showed that the majority of DEGs in rice that responded to SS included starch and sucrose metabolism as well as phenylpropanoid biosynthesis in both cultivars. These defense-responsive pathways were also previously reported as responses to SS in rice, further suggesting that these responses are largely conserved in rice exposed to SS (Wang et al., 2018; Kong et al., 2021). In addition to the pathways listed above, the majority of DEGs in the salt-tolerant cultivar HD961 that responded to SS through transcriptional activation is involved in pathways such as alpha-linolenic acid metabolism, plant hormone signal transduction, plant-pathogen interaction, fatty acid elongation, MAPK signaling pathway–plant, glutathione metabolism, and diterpenoid biosynthesis. Of these pathways, plant hormone signal transduction, MAPK signaling pathway–plant, and glutathione metabolism have been a focus of research on rice undergoing abiotic stress (Kong et al., 2019; Hao et al., 2022).

Several metabolites are changed in plants at different growth stages or when plants are exposed to biotic and abiotic stress, representing the end synthetic products of cells. Liu et al. (2020) postulated that anthocyanins, flavonols, and flavones directly affect the color formation of pepper fruit anthocyanins due to an abundance of flavonols and flavones compared to other varieties. Moreover, the coloration of the red pear fruit results from the induction of ethylene biosynthesis by jasmonate, which decreases anthocyanin production, thus increasing the availability of the precursors for flavone/isoflavone biosynthesis and enhancing the deep yellow fruit coloration (Ni et al., 2020). The abundance of metabolites is most closely related to phenotypes and directly affects plant phenotypes (Fiehn, 2002). In our study, the results suggested that the higher concentration of NaCl (from 0 to 120 mM) resulted in more SRMs. Moreover, rice can synthesize more metabolites to resist damage under salt stress, and we found that most of the metabolites were upregulated. These upregulated SRMs consisted of amino acids and derivatives (20%), organic acids (15.5%), lipids (14.8%), and alkaloids (12.3%), while the downregulated SRMs consisted of flavonoids (25.7%), organic acids (24.5%), and lipids (12.8%). Therefore, amino acids and derivatives were the main upregulated SRMs, whereas flavonoids and organic acids were the main downregulated SRMs in rice under SS. Previous results have shown that sugars and amino acids increased significantly in the leaves and roots of both rice genotypes (FL478 and IR64). In contrast, organic acids increased in roots and decreased in leaves (Zhao et al., 2014). Gupta and De (2017) found that the conserved primary metabolites (sugars, polyols, amino acids, organic acids, and certain purine derivatives) responded differently to NaCl stress in four rice varieties. The SRMs we observed in our study were similar to those previously shown, indicating that the metabolites responding to SS were conserved in rice. Moreover, KEGG pathway enrichment analysis of SRMs showed that the biosynthesis of amino acids was the most significant enrichment in both cultivars under SS among the top 20 KEGG enrichment pathways. Amino acids are the basic substances for synthesizing various metabolites and proteins in plants. Batista-Silva et al. (2019) found a tight relationship between amino acid metabolism and stress responses. We discovered that L-cysteine, L-lysine-butanoic acid, L-valine, L-lysine, and L-proline were all upregulated in these cultivars in response to SS. Previous studies have also shown that proline is a compatible osmolyte during the SS response, alanine and glutamine act as nitrogen stores, and leucine, valine, isoleucine, and lysine are involved with ATP production and detoxification (Hildebrandt, 2018; Liang et al., 2018; Batista-Silva et al., 2019).

This study identified specific metabolic pathways in the HD961 cultivar in response to SS, including glutathione metabolism, ascorbate and aldarate metabolism, and pantothenate and CoA biosynthesis. However, glutathione metabolism was the only pathway that exhibited significant enrichment. Furthermore, the results of the integrative analysis between the transcriptome and metabolome showed that glutathione metabolism was specific and significantly affected in the HD961 cultivar undergoing SS. GSH plays a vital role in plants and regulates various metabolic functions, including antioxidant defense, xenobiotic detoxification, reserving cysteine, maintaining redox balance, immunity modulation, fibrogenesis, cell cycle regulation, and apoptosis (Hasanuzzaman et al., 2017, 2019). Therefore, the glutathione pathway has been a key focus of research on plants undergoing abiotic stress (Khunpon et al., 2018; Wang et al., 2019a,^2021^). Yang et al. (2020) found that the concentration of GSH and GSSG (oxidized glutathione) decreased during seed germination and young seedling growth stages in plants undergoing SS from day zero to four, and the DEGs mainly contained 2 GPX, 3 APX, 1 GSS, and 39 GST. We obtained similar results in this study, whereby GSH decreased in the two cultivars when the concentration of NaCl increased, indicating that a high salt concentration induces rice to produce more peroxides. There were 33 GST DEGs and 13 GST DEGs in the HD961 and 9311 cultivars, respectively, showing that HD961 had a better response to SS than 9311. Moreover, the GSH concentration was positively correlated with *LOC_Os10g38340* (*GSTU19*), *LOC_Os09g37120* (*Ornithine decarboxylase*, *ODC1*), *LOC_Os10g38160* (*GST*), and *LOC_Os01g72130* (*GSTU35*), and negatively correlated with *LOC_Os04g51300* (*APX*), *LOC_Os03g17480* (*GSTL1*), *LOC_Os02g34810* (*APX8*), and *LOC_Os12g16200* (*GSS2*) in HD961, suggesting that these DEGs were candidate salt-tolerance regulation genes. The functions of *OsAPX2* (Zhang et al., 2013), *OsAPX8* (Hong et al., 2007), *OsGSTL2* (Kumar et al., 2013), *OsGR2* (Kaminaka et al., 1998), *OsGR3* (Wu et al., 2015), and *OsGRX1* (Lima-Melo et al., 2016) have been previously identified; the results suggest that these genes are positively or negatively regulated in rice in response to SS. Taken together, the identification of these filtered candidate salt-tolerance regulation genes in this study warrants additional studies to verify their genetic function in the future.

Rice is most sensitive to SS at the seedling and reproductive stages (Ganie et al., 2019). We can improve the salt tolerance of rice in these two stages through water and fertilizer management. Based on the above results, amino acid metabolism and glutathione metabolism actively respond to salt stress during the rice seedling stage. Thus, exogenous application of amino acids on rice leaves is an approach that can enhance the salt tolerance of rice seedlings or appropriate application of nitrogen fertilizer, which has certain reference significance for rice planting and management on saline-alkali land.

## Conclusion

This study found that the growth potential and antioxidant ability of the HD961 cultivar were stronger than those of the cultivar 9311 under SS. Numerous genes and metabolites related to SS were identified through transcriptome and metabolome analysis. Many of the genes and metabolites were common between both cultivars, but HD961 had a higher accumulation and regulation ability, suggesting that this might be the primary driver for salt resistance in HD961. The glutathione metabolism pathway was significantly enriched in HD961 but absent in 9311, and there were more DEGs and SRMs in HD961 than in 9311. These results indicate that glutathione metabolism plays an important role in response to SS and provide a better understanding of the rice regulatory network involved in responding to NaCl.

## Data availability statement

The datasets can be found in an online database. The raw sequence data of RNA-sequencing has been deposited in the NCBI database under Bioproject number: PRJNA842927. The metabolite data reported in this article have been deposited in the OMIX, China National Center for Bioinformation/Beijing Institute of Genomics, Chinese Academy of Sciences (https://ngdc.cncb.ac.cn/omix: accession no. OMIX001300).

## Author contributions

PW, SY, JX, and HZ contributed to the conception and design of the study. ML, NC, and GC organized the database. HG and JM performed the statistical analysis. SY and ML wrote the first draft of the manuscript. JX, NC, GC, and HZ wrote sections of the manuscript. All authors contributed to manuscript revision, read, and approved the submitted version.

## Footnotes

1
http://plants.ensembl.org/Oryza_indica/Info/Index

2
www.metware.cn

3
www.r-project.org

4
http://www.kegg.jp/kegg/compound/

5
http://www.kegg.jp/kegg/pathway.html

6
https://cloud.metware.cn/
