# Supplementary material for: Combined transcriptome and metabolome reveal glutathione metabolism plays a critical role in resistance to salinity in rice landraces HD961
Source: Front Plant Sci. 2022 Sep 7;13:952595. doi: 10.3389/fpls.2022.952595 (PMC9490218; doi:10.3389/fpls.2022.952595)
Supplement: Supplementary file 1 [file Data_Sheet_1.ZIP › Supplementary files/Supplementary figures.docx]

**Combined Transcriptome and Metabolome Reveal Glutathione Metabolism Plays a Critical Role in Resistance to Salinity in Rice Landraces HD961**

Shan Yang^1^, Mengshuang Liu^1^, Na Chu^2^, Guanxiu Chen^1^, Panpan Wang^1^, Junjie Mo^1^, Haifeng Guo^1^, Jianghuan Xu^1^*, Hongkai Zhou^1^*

^1^College of Coastal Agricultural Sciences, South China Branch of National Saline-Alkali Tolerant Rice Technology Innovation Center, Guangdong Ocean University, Zhanjiang, China, 524088

^2^National Engineering Research Center for Sugarcane, Fujian Agriculture and Forestry University, Fuzhou, China, 350002

***Correspondence**Hongkai Zhou
zhouhk@gdou.edu.cn

Jianghuan, Xu

xujianghuan@163.com


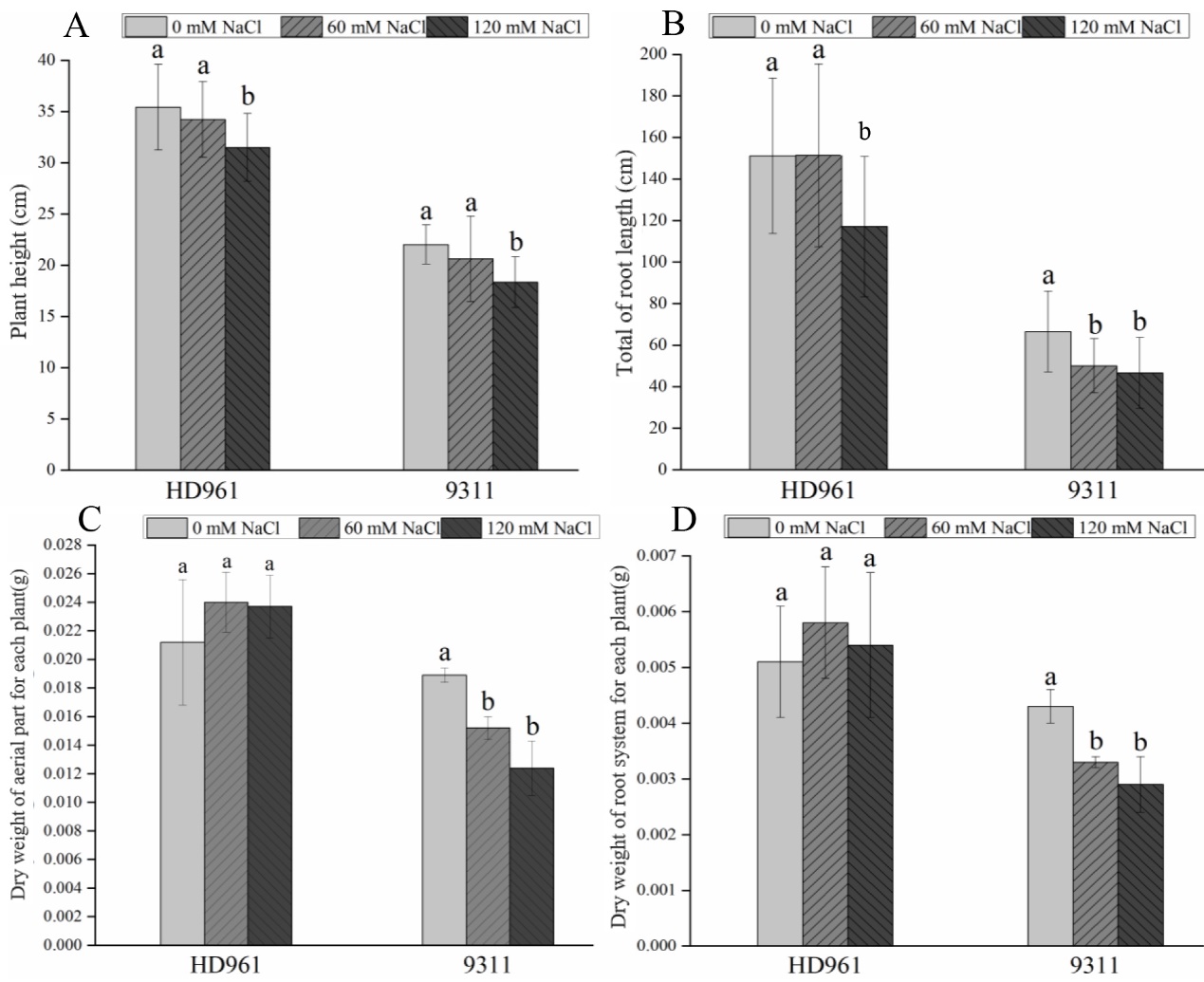


**FIGURE S1** Phenotypic parameters of HD961 and 9311 after 7 d salt stress treatment. (A) Plant height of HD961 and 9311. (B) Root length of HD961 and 9311. (C) Dry weight of above-ground portions of HD961 and 9311. (D) Dry weight of the root of HD961 and 9311. The values of error bars are plus and minus standard deviation values. The different letters denote that it is on the level of significance among different concentrations at the same cultivar, *p* < 0.05.


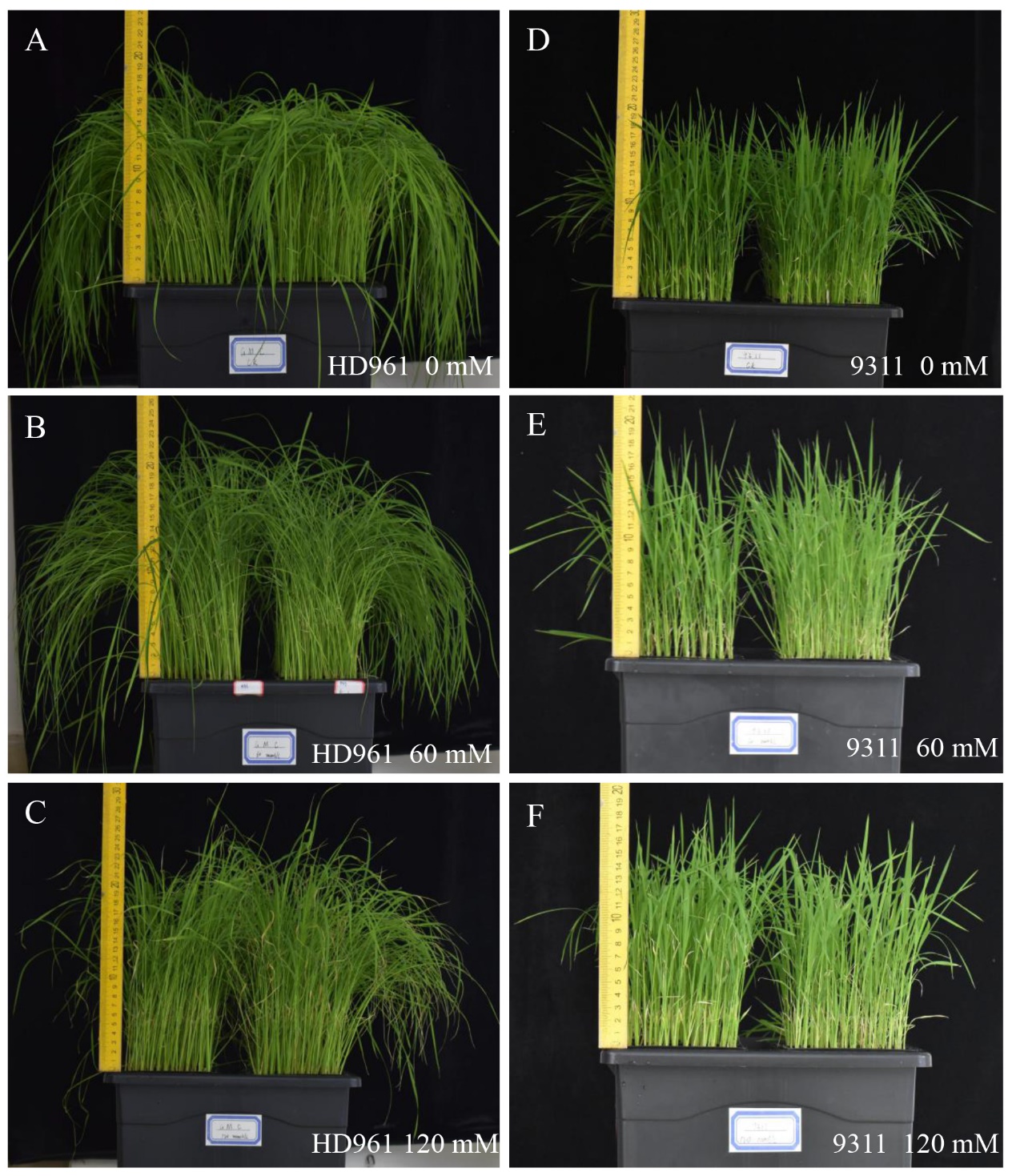


**FIGURE S2** Growth situation of HD961 and 9311 after 7 d salt stress treatment. (A) Growth phenotype of HD961 under 0 mM NaCl treatment. (B) Growth phenotype of HD961 under 60 mM NaCl treatment. (C) Growth phenotype of HD961 under 120 mM NaCl treatment. (D) Growth phenotype of 9311 under 0 mM NaCl treatment. (E) Growth phenotype of 9311 under 60 mM NaCl treatment. (F) Growth phenotype of 9311 under 120 mM NaCl treatment.


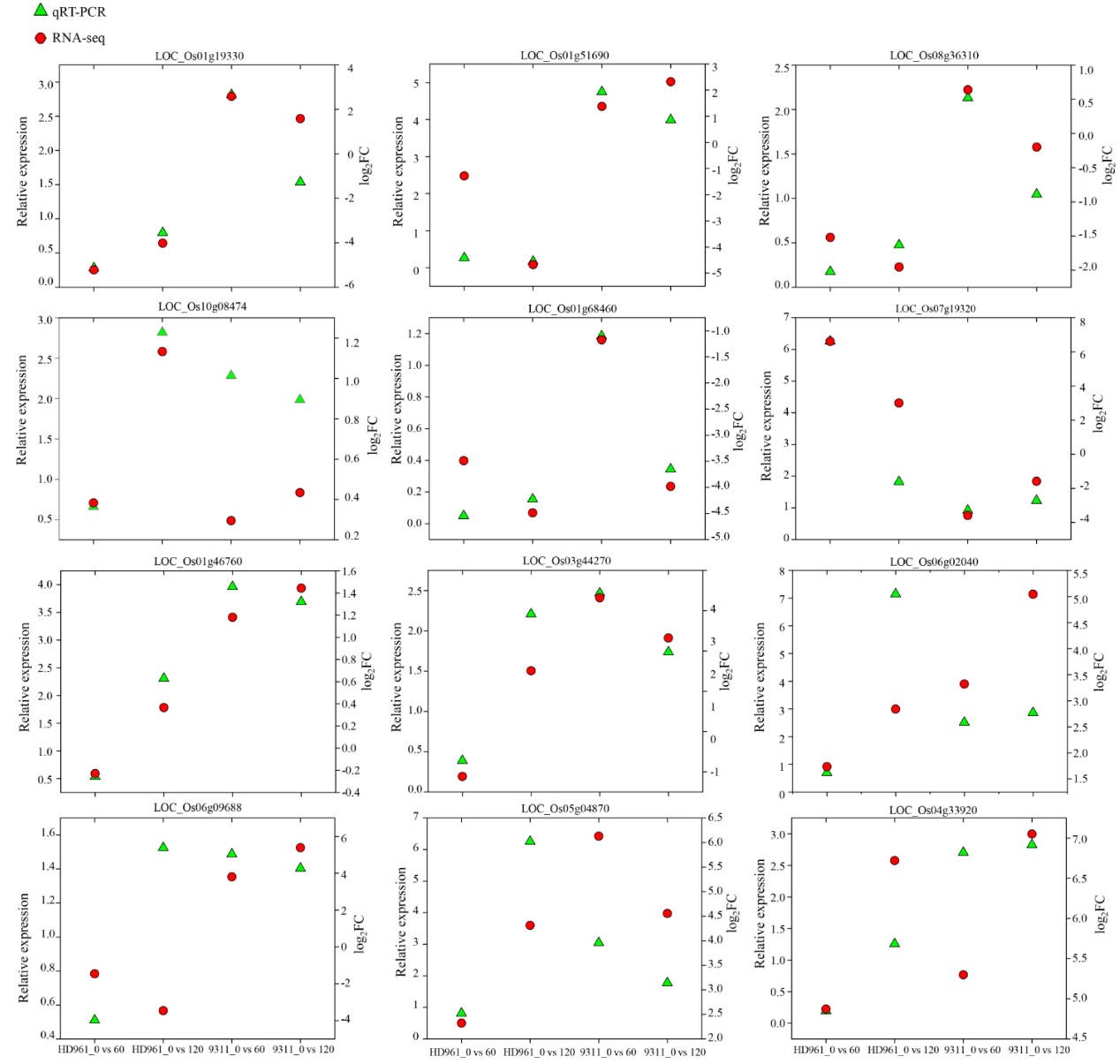


**FIGURE S3** The results of qRT-PCR and RNA-seq of 12 genes in the four groups.


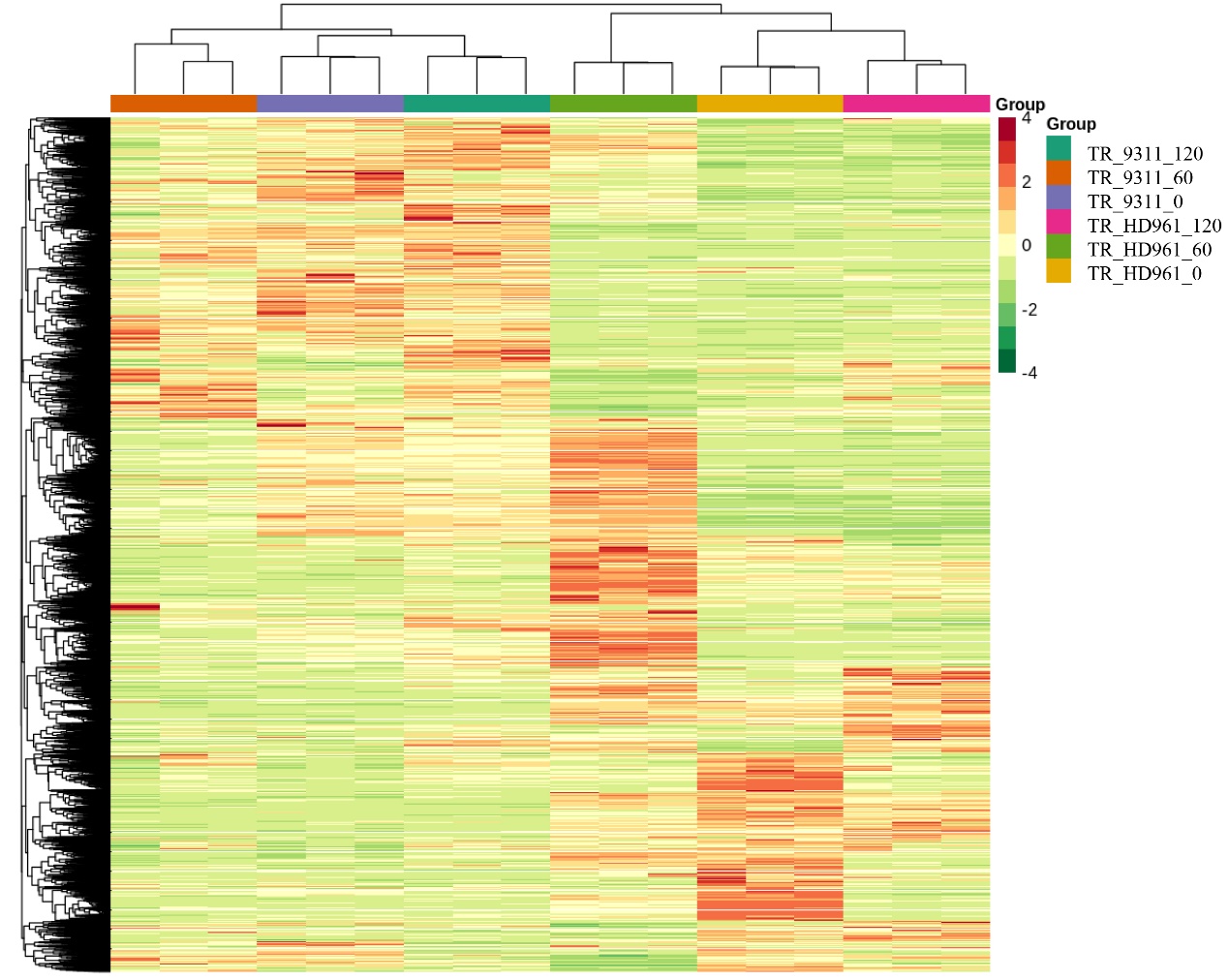


**FIGURE S4** Heatmap hierarchical clustering of all detected genes.


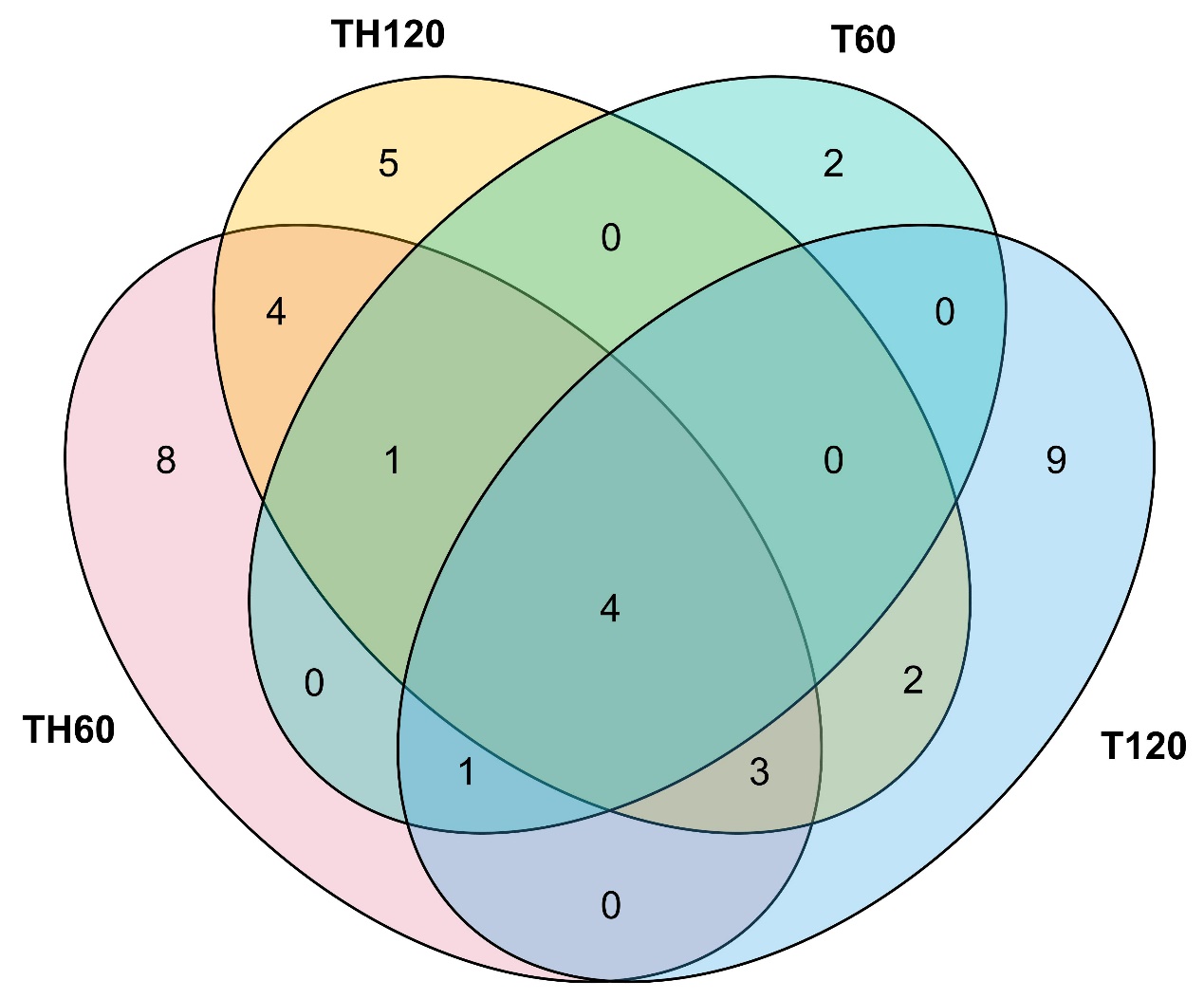


**FIGURE S5** Venn diagram of significantly different KEGG pathways of DEGs in HD961 and 9311.


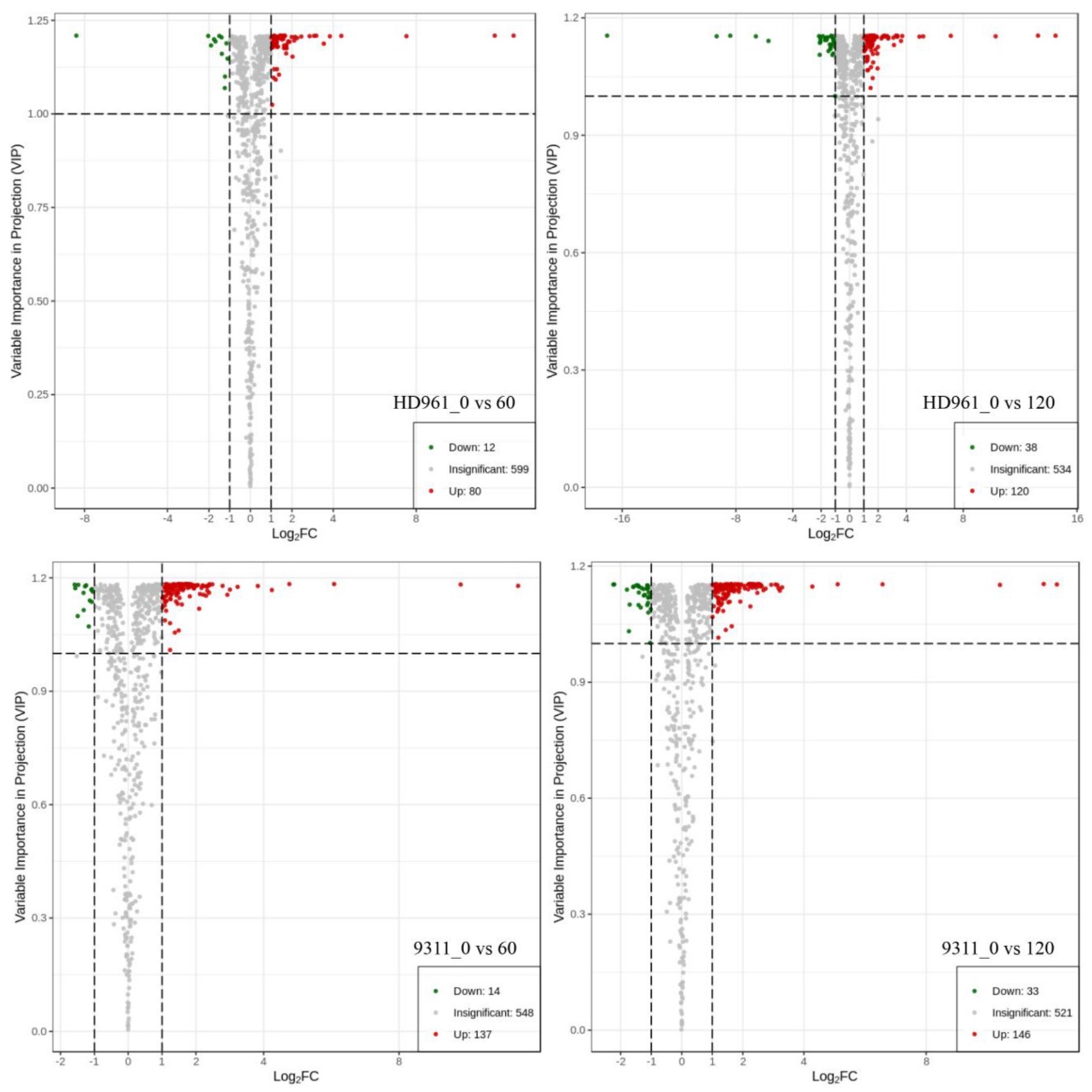


**FIGURE S6** Volcano plot of SRMs in the HD961 and 9311.


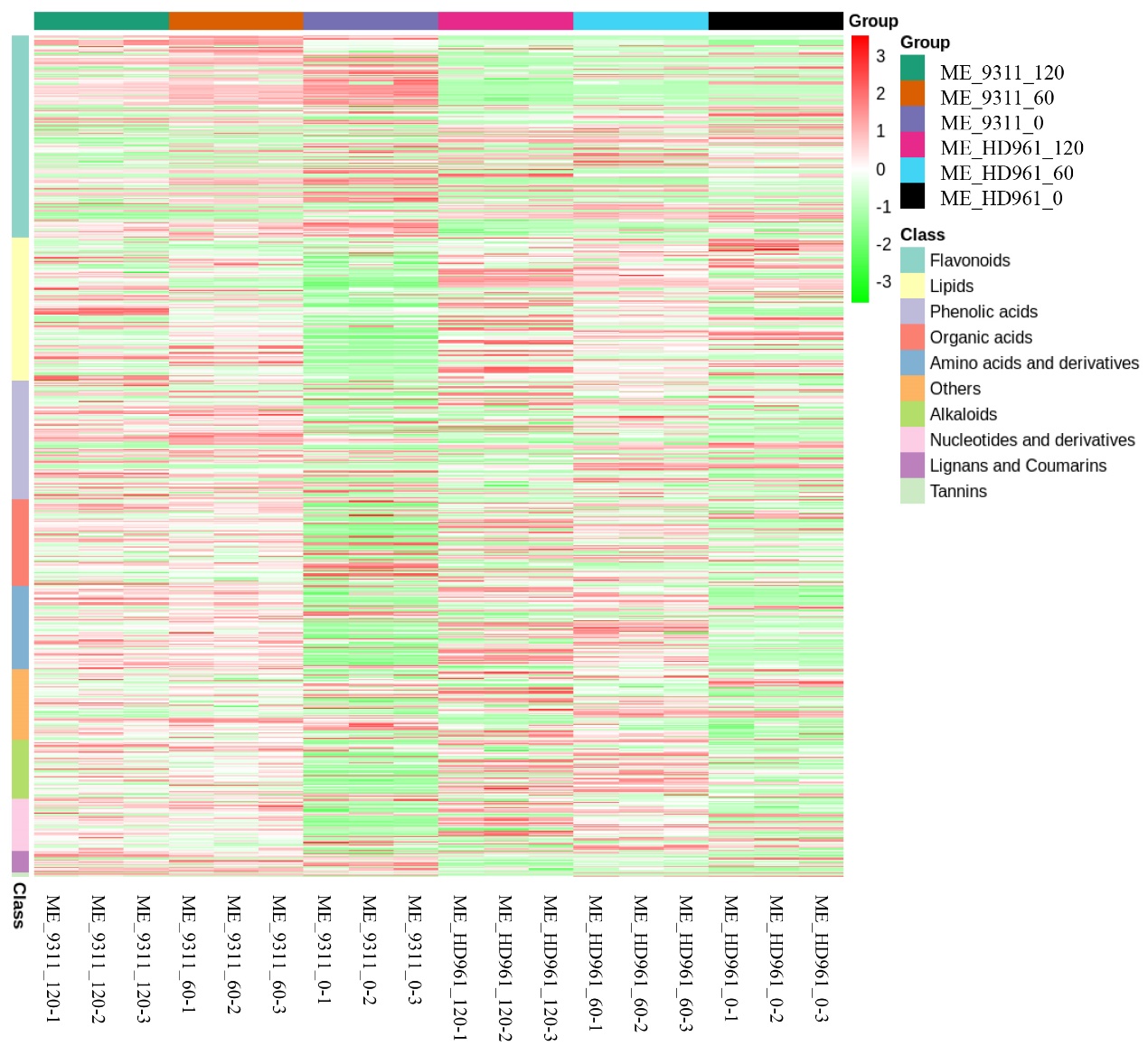


**FIGURE S7** Heatmap hierarchical clustering of all detected metabolites.


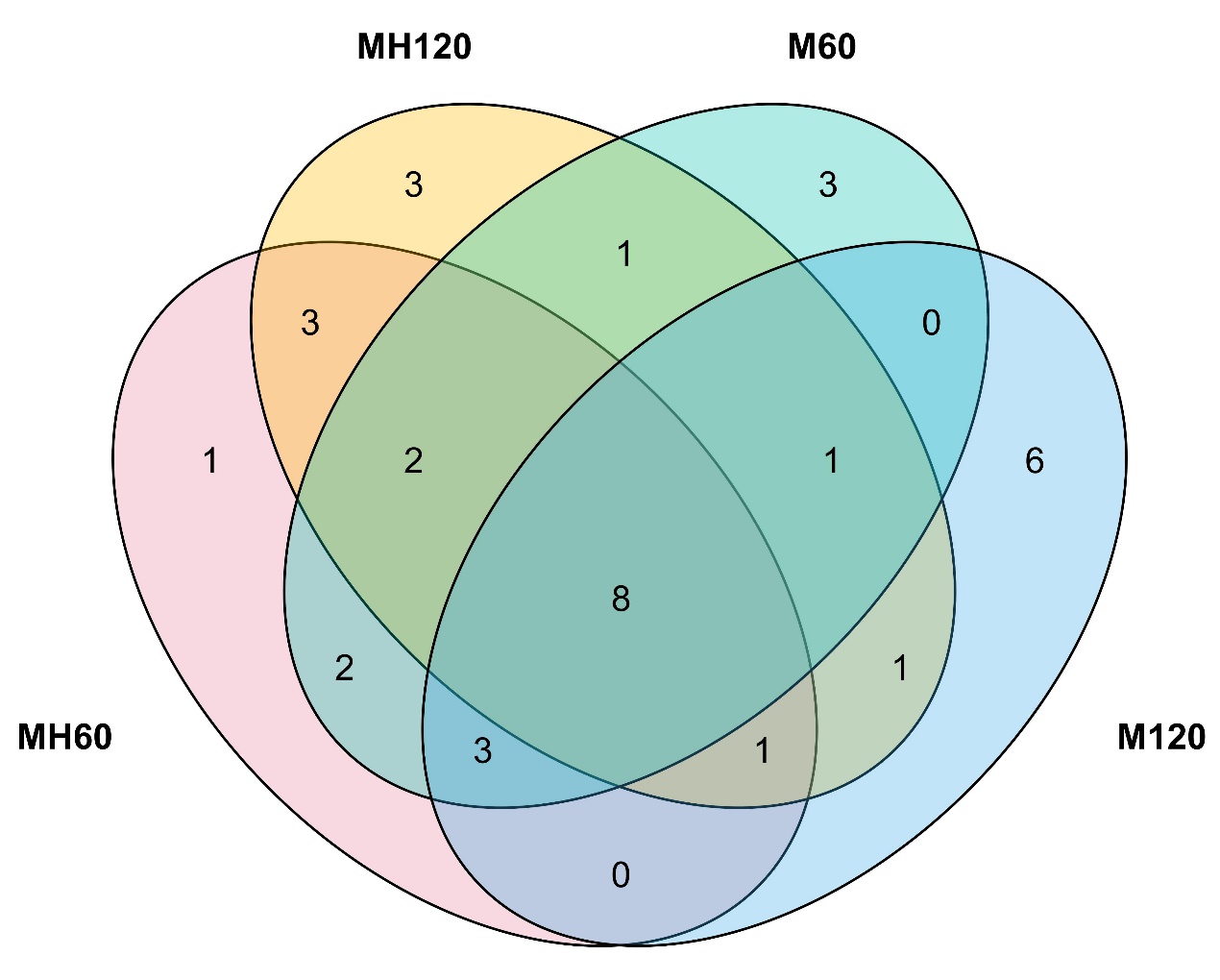


**FIGURE S8** Venn diagram of top-20 KEGG enrichment pathway of SRMs in HD961 and 9311.
