# Supplementary material for: Combined transcriptome and metabolome reveal glutathione metabolism plays a critical role in resistance to salinity in rice landraces HD961
Source: Front Plant Sci. 2022 Sep 7;13:952595. doi: 10.3389/fpls.2022.952595 (PMC9490218; doi:10.3389/fpls.2022.952595)
Supplement: Supplementary file 1 [file Data_Sheet_1.ZIP › Supplementary files/Supplementary table legends.docx]

**Combined Transcriptome and Metabolome Reveal Glutathione Metabolism Plays a Critical Role in Resistance to Salinity in Rice Landraces HD961**

Shan Yang^1^, Mengshuang Liu^1^, Na Chu^2^, Guanxiu Chen^1^, Panpan Wang^1^, Junjie Mo^1^, Haifeng Guo^1^, Jianghuan Xu^1^*, Hongkai Zhou^1^*

^1^College of Coastal Agricultural Sciences, South China Branch of National Saline-Alkali Tolerant Rice Technology Innovation Center, Guangdong Ocean University, Zhanjiang, China, 524088

^2^National Engineering Research Center for Sugarcane, Fujian Agriculture and Forestry University, Fuzhou, China, 350002

***Correspondence**Hongkai Zhou
zhouhk@gdou.edu.cn

Jianghuan, Xu

xujianghuan@163.com

TABLE S1 Primers of genes

TABLE S2 Summary of RNA-seq data quality

TABLE S3 KEGG enrichment of DEGs in the TH60.

TABLE S4 KEGG enrichment of DEGs in the TH120.

TABLE S5 KEGG enrichment of DEGs in the T60.

TABLE S6 KEGG enrichment of DEGs in the T120.

TABLE S7 Top-10 up-regulated and down-regulated DAMs of HD961..

TABLE S8 Top-10 up-regulated and down-regulated of 9311.

TABLE S9 KEGG enrichment of DAMs in the MH60.

TABLE S10 KEGG enrichment of DAMs in the MH120.

TABLE S11 KEGG enrichment of DAMs in the M60.

TABLE S12 KEGG enrichment of DAMs in the M120.

TABLE S13 The similarities and differences of top-20 KEGG enrichment pathways in four groups.

TABLE S14 Integrative analysis between transcriptome and metabolome in the group HD961_0_vs_60.

TABLE S15 Integrative analysis between transcriptome and metabolome in the group HD961_0_vs_120.

TABLE S16 Integrative analysis between transcriptome and metabolome in the group 9311_0_vs_60.

TABLE S17 Integrative analysis between transcriptome and metabolome in the group 9311_0_vs_120.
